# High-Grade Appendiceal Goblet Cell Adenocarcinoma—A Literature Review Starting from a Rare Case

**DOI:** 10.3390/life15071047

**Published:** 2025-06-30

**Authors:** Mircea Gheorghe, Rodica Daniela Birla, Anca Evsei-Seceleanu, Luiza Bitina, Ioan Nicolae Mates, Dragos Valentin Predescu

**Affiliations:** 1 General and Esophageal Surgery Clinic, Sfanta Maria Clinical Hospital, Carol Davila University of Medicine and Pharmacy, 020021 Bucharest, Romania; mircea.gheorghe@umfcd.ro (M.G.); dragos.predescu@umfcd.ro (D.V.P.); 2 Department of Pathology, Sfanta Maria Clinical Hospital, Carol Davila University of Medicine and Pharmacy, 011172 Bucharest, Romania; anca.evsei@umfcd.ro; 3 Sfantul Nectarie Oncological Center, 200745 Craiova, Romania; bitinaluiza@gmail.com

**Keywords:** gobletcell adenocarcinoma, appendiceal, review, rare case report, current management

## Abstract

Goblet cell adenocarcinomas (GCAs) are an exceedingly rare subtype of tumors, almost exclusively occurring in the appendix, and characterized by features overlapping both adenocarcinomas and neuroendocrine tumors (NETs), which has historically led to confusion and varied nomenclature. This study presents a comprehensive review of the literature highlighting particularities of this type of malignancy, starting from a rare case of a 54-year-old female operated on in our clinic for an appendiceal tumor, initially suspected to be a mucinous neoplasm based on colonoscopic biopsy, which was ultimately confirmed to be goblet cell adenocarcinoma on both intraoperative frozen section and definitive pathological examination. Exhibiting signs and symptoms associated with an abdominal mass, she underwent a right hemicolectomy with partial omentectomy for locally advanced, high-grade, invasive goblet cell adenocarcinoma of the appendix with lymphatic macro metastases and epiploic invasion, categorized as AJCC stage IVb carcinomatosis. The patient received FOLFOX adjuvant. Six months later, she required reoperation due to the progression of carcinomatosis, which was again confirmed histopathologically. A second-line oncological protocol comprising irinotecan, capecitabine, and bevacizumab was initiated. Given the rarity of GCAs and the absence of a consensus on nomenclature, classification, and diagnostic criteria, we conducted a comprehensive literature review to highlight current trends related to this entity, including its classification within different systems (Tang, Yozu, WHO, AJCC), as well as the therapeutic surgical approaches—ranging from simple appendectomy to extensive multiorgan resection, cytoreductive surgery (CRS) combined with hyperthermic intraperitoneal chemotherapy (HIPEC), and the use of systemic therapy. Adhering to these recommendations will enhance communication among pathologists, surgeons, and oncologists regarding the natural history and prognosis of this rare malignancy.

## 1. Introduction

Cancers and tumors of the appendix encompass a wide histological spectrum, as outlined in the WHO Classification of Tumors. Following neuroendocrine tumors (NETs), the second most common malignancy of the appendix is adenocarcinoma. Appendiceal goblet cell adenocarcinomas (AGCAs) represent an exceptionally rare subtype, with an incidence of approximately 0.05 per 100,000 individuals per year in North America. Unlike conventional adenocarcinomas, AGCAs exhibit features of both adenocarcinomas and NETs, a duality that has historically led to multiple terminologies and some degree of diagnostic confusion [1]. This tumor type accounts for less than 14% of all appendiceal neoplasms [2].

GCAs show a degree of neuroendocrine differentiation, characterized by the expression of chromogranin A and B, synaptophysin, and allelic losses on chromosomes 11q, 16q, and 18q—features that mirror those seen in neuroendocrine neoplasms (NENs) of the jejunum and ileum [3]. Notably, the Ki-67 proliferation index in GCAs is significantly higher than in intestinal NENs [4]. Immunohistochemically, GCAs can be distinguished from mucin-producing NENs by their stronger expression of carcinoembryonic antigen (CEA), CDX-2, CAM5.2, and cytokeratins (CK), particularly when compared to NENs [5]. Furthermore, the reduced expression of neuroendocrine markers has been observed in metastatic sites in some patients with GCA [6].

Although goblet cell adenocarcinomas (GCA) contain neuroendocrine cells—supporting their historical designation as “carcinoid” tumors and prompting classification and treatment approaches similar to neuroendocrine tumors (NETs)—in clinical practice they behave more like appendiceal adenocarcinomas. Consequently, they require staging and treatment strategies aligned with adenocarcinomas rather than NETs. To avoid this diagnostic and therapeutic confusion, the World Health Organization (WHO) reclassified these tumors as goblet cell adenocarcinoma in its 2019 classification [1].

Despite their rarity, these tumors can be encountered in clinical practice and must be recognized and managed with appropriate urgency, from initial diagnosis to multimodal therapeutic intervention. Currently, GCAs are staged and treated according to adenocarcinoma protocols due to their more aggressive biological behavior, though treatment approaches remain non-standardized.

GCA exhibits significantly greater malignant potential than NENs, necessitating clear diagnostic separation [7,8]. This clinical divergence is reflected in disease-specific five-year survival rates, which exceed 90% for appendiceal NENs but range from 58% to 81% for GCA [9,10,11].

In this article, we present a case of advanced AGCA treated surgically and offer a series of reflections based on a review of the literature, highlighting the tumor’s rarity, unresolved aspects of its histogenesis, terminological inconsistencies, and the implications these issues have on classification and treatment.

## 2. Case Presentation

A 54-year-old female presented to the emergency department with a palpable mass in the right iliac fossa. She reported no localized or generalized abdominal pain, weight loss, or other systemic symptoms. Her medical history included bronchial asthma and chronic obstructive pulmonary disease (COPD), for which she was on Symbicort, Montelukast, and Aerius. She also had autoimmune thyroiditis managed with Euthyroxand a history of two neurovascular strokes two years prior. Family history was notable for hypertension and ischemic heart disease in both parents.

On physical examination, the patient was obese (BMI: 35.11 kg/m^2^) and hemodynamically stable, with a blood pressure of 127/77 mmHg, pulse rate of 81 beats per minute, respiratory rate of 18 breaths per minute, oral temperature of 36.9 °C, and oxygen saturation of 97%. Abdominal examination revealed a soft, non-tender abdomen with a palpable mass in the right lower quadrant, without rebound tenderness or guarding. Cardiopulmonary examination was unremarkable.

Laboratory investigations were within normal limits, including inflammatory markers, complete blood count, coagulation profile, and renal and liver function tests. Tumor markers showed CA19-9 at 25.9 U/mL (reference range: 0–33 U/mL) and carcinoembryonic antigen (CEA) at 2.57 ng/mL.

**Figure 1:**
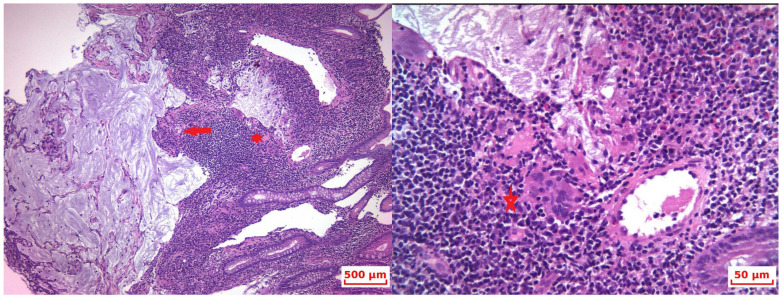
HP—Colonic mucosa with acellular mucus islets (red arrow) and chronic granulomatous inflammation with multinucleated “foreign body” giant cells arranged around the mucin (red star). (HE 10×and detail).

Upper gastrointestinal endoscopy revealed no abnormalities in the esophagus, stomach, or duodenum. Colonoscopy was unremarkable except for a 0.5 cm cecal invagination at the appendiceal orifice, with macroscopically normal mucosa. Biopsied cecal mucosa revealed only submucosal mucin pools, without any other associated lesions raising suspicion for a mucinous neoplasm invading from the outside (Figure 1).

**Figure 2:**
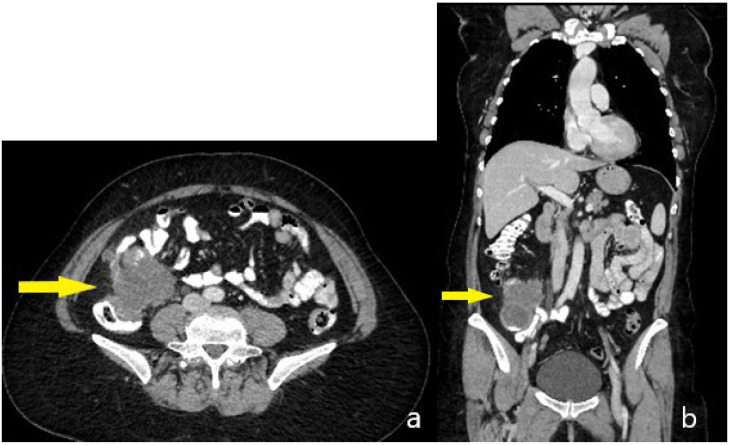
Preoperative CT scan—(**a**) Transversal aspect, tumor–arrow, (**b**) Sagittal aspect, tumor–arrow, no distant visible metastases.

Contrast-enhanced computed tomography (CT) of the thorax, abdomen, and pelvis demonstrated a 58 × 58 × 74 mm mass in the appendix, adherent to the cecum and distal ileum without clear demarcation, accompanied by dense periappendiceal fat stranding and regional lymph nodes measuring up to 15 × 13 mm. No evidence of pulmonary, hepatic, or peritoneal metastases was observed (Figure 2a,b).

Cardiologic evaluation yielded a Lee index score of 1, with an estimated functional capacity exceeding four metabolic equivalents (METs) and a left ventricular ejection fraction of 60%. Pulmonary assessment, considering the patient’s COPD history, indicated eosinophilic asthma, classified as Grade III according to the Global Initiative for Asthma (GINA) guidelines, with documented allergies to ampicillin and dust mites. Spirometry revealed a forced expiratory volume in one second (FEV1) of 94% and an inspiratory time (IT) of 76.6%.

**Figure 3:**
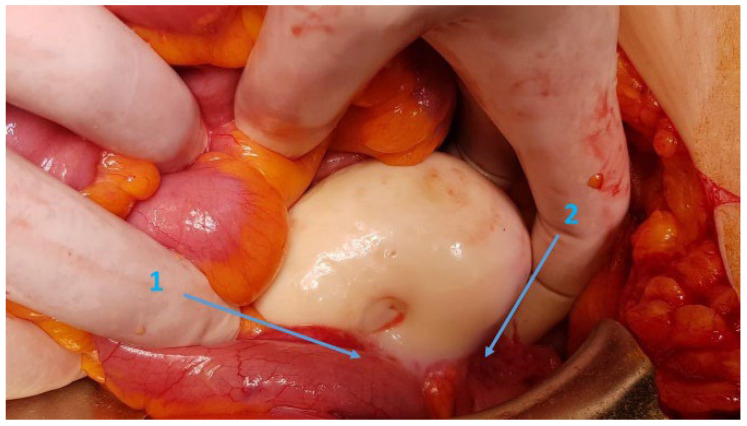
Intraoperative aspect of appendiceal tumor invading distal ileum (1) and cecum (2).

Following multidisciplinary oncologic and surgical team discussions, the patient underwent open right ileo-hemicolectomy, right adnexectomy, and omentectomy under general anesthesia. Intraoperatively, a firm, white, 7 × 7 cm mucinous tumor originating from the appendix was identified, invading the cecum and distal ileum, (Figure 3) with peritoneal dissemination involving nodules less than 0.5 mm in diameter.

A side-to-side ileocolic anastomosis was performed. The total operative time was 230 min, with minimal blood loss and no transfusion requirements.

The postoperative course was uneventful, with the return of bowel function by postoperative day 2 and tolerance of oral intake by day 3. A minor thromboembolism was suspected on postoperative day 6 but was ruled out by pulmonary CT angiography. The patient was discharged on postoperative day 8, with a total hospital stay of 11 days.

**Figure 4:**
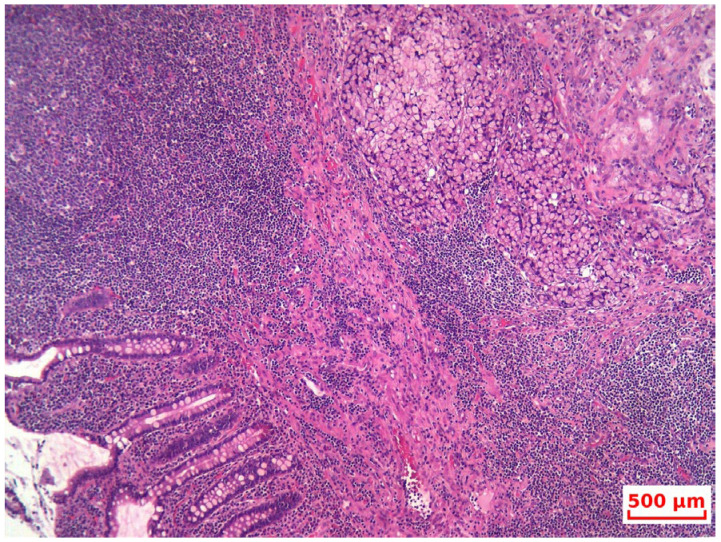
HP—At low magnification, the tumor is seen infiltrating normal colonic glands, as nests and small, rounded clusters of cells, many of which are distended by mucin (HE, 10×).

**Figure 5:**
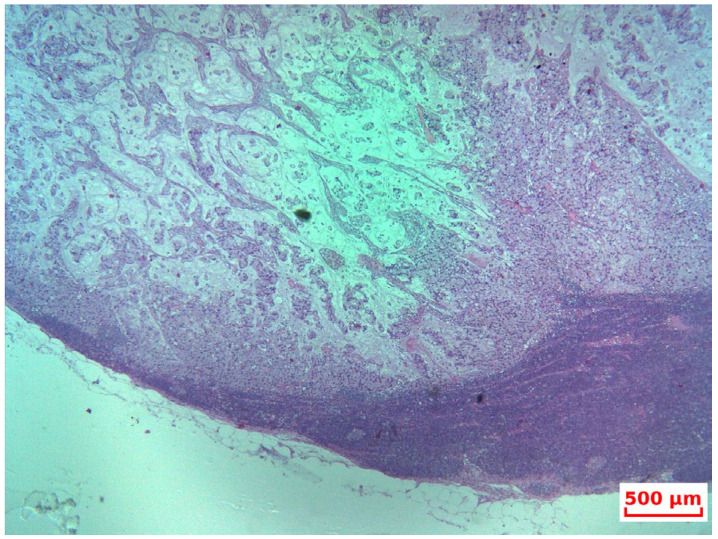
Lymph node with tumor metastasis (HE, 4×).

Final histopathological examination confirmed a high-grade goblet cell adenocarcinoma of the appendix, invading the submucosa of the cecum, with pericecal lymph node metastases (2 out of 29 nodes) and omental involvement, consistent with extensive peritoneal carcinomatosis (Figure 4 and Figure 5).

**Figure 6:**
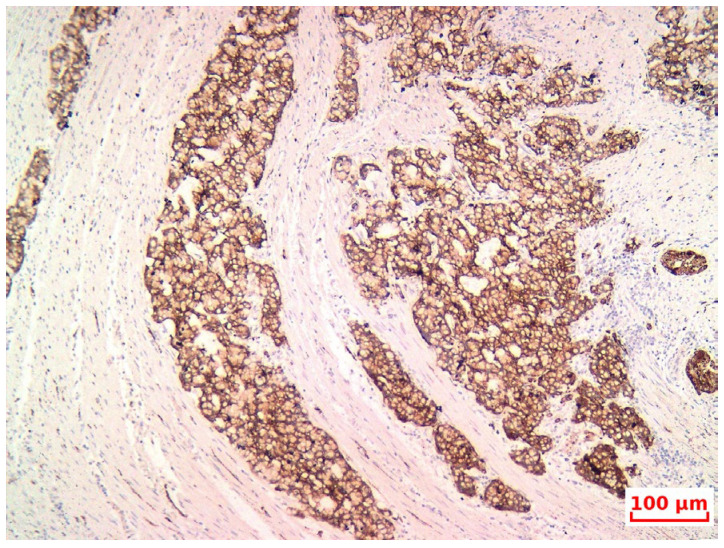
IHC—scattered peripheral endocrine cells within the tumor nests, Synaptophisin, 20×.

**Figure 7:**
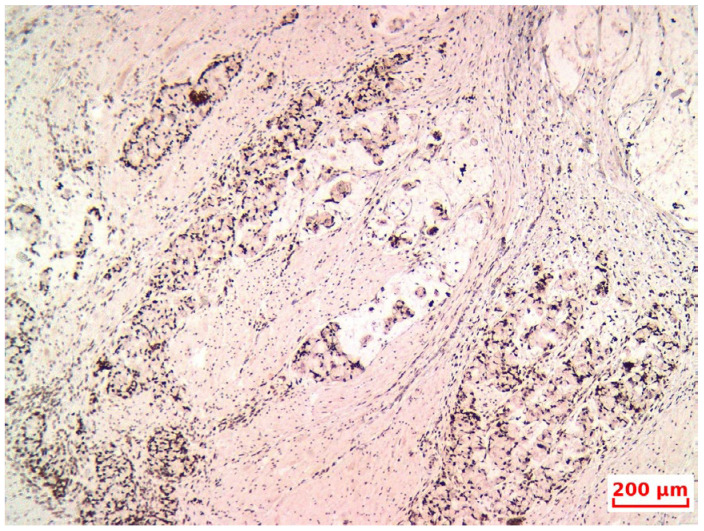
IHC MLH1, 20×.

The tumor was staged as pT4b N1b M1b, with positive lymphovascular and perineural invasion, corresponding to stage IVb according to the AJCC classification. Immunohistochemical analysis revealed positivity for synaptophysin and chromogranin A (Figure 6) neuroendocrine cells scattered through the goblet-like mucinous cells, indicating amphicrine differentiation. Mismatch repair protein expression was intact (Figure 7).

Exon 2 KRAS mutation was identified by molecular biology testing.

**Figure 8:**
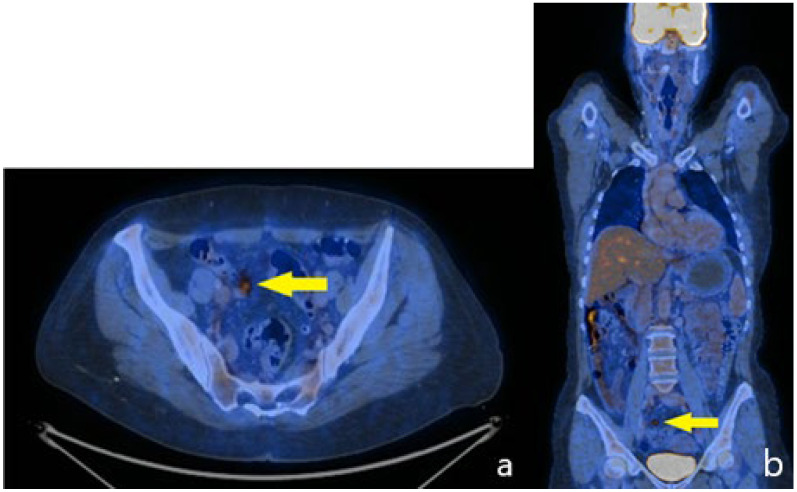
PET CT—postoperative aspect at 6 months, one hot nodule on the right iliac vessels—arrow (**a**) Transversal view; (**b**) Coronal view.

Adjuvant chemotherapy following colorectal cancer protocols was initiated, consisting of 12 cycles of FOLFOX (5-fluorouracil, leucovorin, and oxaliplatin). This regimen is supported by retrospective series and expert consensus, especially in node-positive appendiceal adenocarcinomas, where fluoropyrimidine–oxaliplatin-based chemotherapy has shown a potential survival benefit. At six months postoperatively, positron emission tomography–computed tomography (PET-CT) identified a hypermetabolic nodule adjacent to the right common iliac vein. (Figure 8a,b) (routinely PET-CT scan—during oncological follow-up). Surgical resection of this lesion and additional peritoneal nodules confirmed recurrent goblet cell adenocarcinoma.

Second-line chemotherapy with irinotecan, capecitabine, and bevacizumab was started and well tolerated.

Ongoing surveillance includes clinical evaluations and serum CEA measurements every three months, with contrast-enhanced CT scans of the chest, abdomen, and pelvis every six months for the first two years, followed by annual assessments up to five years. The patient is currently nine months’ post-reoperation, with a follow-up colonoscopy scheduled at one year postoperatively.

## 3. Literature Review

Appendiceal Goblet Cell Adenocarcinoma (AGCA), historically referred to as goblet cell carcinoid (GCC), is a rare and aggressive neoplasm exhibiting both glandular and neuroendocrine differentiation. First described by Gagné in 1969, AGCA has been the subject of numerous case reports and series, reflecting its rarity and the evolving understanding of its pathogenesis and clinical behavior management.

This paper presents the key literature that has contributed to the current understanding and clarification of the histopathological diagnosis, as well as the therapeutic management of the disease in accordance with current clinical guidelines.

A targeted literature search was conducted using the PubMed database to identify relevant publications addressing the histopathological classification and therapeutic management of appendiceal goblet cell adenocarcinoma (AGCA). (Table S1).

The search strategy employed specific keywords and Boolean operators across academic search engines, using the following phrases: “appendiceal goblet cell adenocarcinoma diagnosis” AND “appendiceal goblet cell adenocarcinoma treatment” AND “appendiceal goblet cell adenocarcinoma diagnosis and treatment”. Filters applied to the search included: language (English), age group (adults aged ≥ 19 years), and publication date range (2015–2025). The search strategy plan can be visualized in Supplementary Material Table S1.

A total of 108 and, respectively, 92 articles were screened for diagnosis and, respectively, clinical treatment and outcomes aspects. After two stages of exclusion and selection, see the PRISMA flow charts, two groups were selected for review and grouped in the Supplementary Section as follows: Figure S1—diagnostic studies and Figure S2—clinical, treatment, and outcomes. In the diagnosis table we added 10 reports from external websites and citations in our manuscript, and one report in the treatment table (Table 1 and Table 2).

**Table 1:** Included reports in diagnosis challenges.

No	Authors	Year	Title	Number of Cases
1	Clift et al. [8]	2018	Goblet cell carcinoid of the appendix—diagnostic challenges and treatment updates: a case report and review of the literature.	21 Tang ABC TNM
2	Rossi et al. [12]	2022	Goblet Cell Adenocarcinoma of the Appendix: A Systematic Review and Incidence and Survival of 1225 Cases From an English Cancer Registry.	1225 Tang TNM
3	Reid Y et al. [13]	2022	Appendiceal Goblet Cell Adenocarcinoma: A Historically Informed Reading of 6 Cases.	6 TNM retrospective
4	Taggart et al. [14]	2024	Goblet Cell Carcinoid of the Appendix: Six Case Reports.	6 TNM
5	Lamarca et al. [15]	2018	Adenocarcinoma Ex-Goblet Cell: A Retrospective Experience.	23 Tang B, C
6	Nonaka et al. [16]	2015	Goblet cell appendiceal tumors—management dilemmas and long-term outcomes.	48 Tang ABC
7	Falk et al. [17]	2023	Clinicopathological spectrums and prognosis of primary appendiceal adenocarcinoma, goblet cell adenocarcinoma, and low-grade appendiceal mucinous neoplasms.	40 G1, G2, G3
8	Ibrahim et al. [18]	2020	Clinicopathological analysis of appendiceal goblet cell adenocarcinoma with peritoneal metastasis: World Health Organization grade predicts survival following cytoreductive surgery with intraperitoneal chemotherapy.	63 G1, 2, 3 Tang ABC
9	Gilmore et al. [19]	2020	Molecular Characterization of Appendiceal Goblet Cell Carcinoid.	53 NGS TP53-24.0% ARID1A-15.4% SMAD4-9.4% KRAS-7.5% MSI, MMR-0% PDL1-2%
10	Olsen et al. [20]	2016	Adenocarcinoma ex-goblet cell carcinoid (appendiceal-type crypt cell adenocarcinoma) is a morphologically distinct entity with highly aggressive behavior and frequent association with peritoneal/intra-abdominal dissemination: an analysis of 77 cases.	77 High grade AexGCC
11	Tsang et al. [21]	2018	A study of appendiceal crypt cell adenocarcinoma (so-called goblet cell carcinoid and its related adenocarcinoma).	105 TNM Low grade, high grade
12	Yozu et al. [22]	2015	Goblet cell carcinoid tumor, mixed goblet cell carcinoid-adenocarcinoma, and adenocarcinoma of the appendix: comparison of clinicopathologic features and prognosis.	74 TNM
13	Zakka et al. [23]	2016	Appendiceal Goblet Cell Carcinoids: Management Considerations from a Reference Peritoneal Tumour Service Centre and ENETS Centre of Excellence.	74 TNM AJCC Tang
14	Webb et al. [24]	2018	Histologic and Outcome Study Supports Reclassifying Appendiceal Goblet Cell Carcinoids as Goblet Cell Adenocarcinomas, and Grading and Staging Similarly to Colonic Adenocarcinomas.	126 Low grade High grade TNM AJCC
15	Wang et al. [25]	2021	Is adjuvant chemotherapy beneficial for stage II–III goblet cell carcinoid/goblet cell adenocarcinoma of the appendix?	619 AJCC
16	Jedrzkiewicz et al. [26]	2018	Endoscopic diagnosis of a goblet cell carcinoid tumour of the appendix.	1 TNM
17	Chen et al. [27]	2020	Outcomes in Peritoneal Carcinomatosis from Appendiceal Goblet Cell Carcinoma Treated with Cytoreductive Surgery and Hyperthermic Intraperitoneal Chemotherapy (CRS/HIPEC).	27 TNM AJCC
18	Arai et al. [28]	2018	Adenocarcinoma Ex-goblet Cell Carcinoid of the Appendix: a Case Report and Overview of the Disease.	1 Grade B
19	Taniguchi et al. [29]	2020	Impact of Referral Center Pathology Review on Diagnosis and Management of Patients With Appendiceal Neoplasms.	24 NS
20	Kowalsky et al. [30]	2015	Goblet cell carcinoids: characteristics of a Danish cohort of 83 patients.	83 TNM, Tang
21	Zambrano et al. [31]	2018	Goblet cell carcinoid of the appendix—diagnostic challenges and treatment.	1 TNM, Tang
22	Madsen et al. [32]	2021	Appendiceal tumors with glandular and neuroendocrine features exhibiting peritoneal metastases—Critical evaluation of outcome following cytoreductive surgery with perioperative chemotherapy.	47 TNM, Tang
23	Radomski et al. [33]	2021	Lymph node positivity and association with long-term survival for different histologies of appendiceal cancer.	4435 TNM
24	Das et al. [34]	2019	Treatment patterns and outcomes in goblet cell carcinoid tumors of the appendix.	2552 AJCC, G
25	Barrak et al. [35]	2025	Impact of genetic mutations on prognosis and chemotherapy efficacy in advanced appendiceal carcinoma: insights from the nationwide Japanese comprehensive genomic profiling test database.	39 Gene panel testing
26	Shyu et al. [36]	2021	Chemotherapy in the treatment of different histological types of appendiceal cancers: a SEER based study.	1087 TNM AJCC
27	Palmer et al. [37]	2018	Outcomes of Surgical and Chemotherapeutic Treatments of Goblet Cell Carcinoid Tumors of the Appendix.	86/88 TNM, Tang
28	Höfler et al. [38]	2018	Effects of Cytoreductive Surgery and Hyperthermic Intraperitoneal Chemotherapy (HIPEC) in the Treatment of Goblet Cell Carcinoma: A Prospective Cohort Study.	48 TNM

### 3.1. Diagnosis Challenges

Notably, 29 of the included diagnostic articles (21 from databases and eight from external sources) were published after 2019, the year in which the World Health Organization (WHO) introduced the current classification for appendiceal tumors. This updated terminology formally recommends the use of “Appendiceal Goblet Cell Adenocarcinoma (AGCA)” to ensure accurate pathological classification.

Earlier nomenclature used prior to 2019, such as *appendiceal goblet cell carcinoid*, *adenocarcinoma ex goblet cell carcinoid* and *adenocarcinoid*, was also included in the discussion due to the presence of large retrospective series published under these historical terms.

Some authors propose that goblet cell carcinoma (GCC) is best regarded as a distinct variant of adenocarcinoma, suggesting that the term “crypt cell adenocarcinoma” more accurately reflects both the cellular origin and histopathological nature of this tumor entity.

The study by Rossi et al. involving 48 cases of appendiceal goblet cell tumors (GCTs)—stratified by stage as follows: TNM stage I–II: 27 cases, stage III: 15 cases, and stage IV: six cases—demonstrated that GCTs exhibit a more aggressive clinical course and are associated with poorer outcomes compared to midgut neuroendocrine tumors (mNETs). The authors concluded that the clinical behavior of GCTs more closely resembles that of colorectal adenocarcinomas rather than that of classical neuroendocrine tumors, highlighting the need for distinct diagnostic and therapeutic approaches [12].

In a comprehensive study by Reid et al., analyzing 77 cases of appendiceal adenocarcinoma ex-goblet cell carcinoid (also referred to as appendiceal-type crypt cell adenocarcinoma), the authors concluded that this neoplasm represents a morphologically distinct entity characterized by highly aggressive clinical behavior and a marked tendency for peritoneal and intra-abdominal dissemination. The tumors demonstrated a wide spectrum of histologic patterns, often occurring in variable combinations [13].

In a study of 74 cases, Taggart et al. emphasized the importance of subclassifying appendiceal tumors with goblet cell tumor (GCT) morphology based on the proportion of adenocarcinoma components, as this histologic detail was shown to correlate significantly with disease stage and had a direct impact on overall survival [14].

In a separate single-centre retrospective study also analyzing 74 cases, Lamarca et al. retained the use of the term goblet cell carcinoma (GCC) and investigated the prognostic impact of age and Tang subclassification on overall survival (OS), highlighting the relevance of tumor grading and patient-related factors in outcome prediction [15].

In the study conducted by Nonaka et al., which analyzed 105 cases, approximately one third of the tumors were purely low-grade, while the remaining cases exhibited a variable high-grade component, ranging from 5% to 95%. The neuroendocrine cell component ranged from 0% to 90% (median: 5%), whereas, the mucinous cell component ranged from 5% to 100% (median: 70%). Importantly, there was a statistically significant difference in cancer-specific survival (CSS) when tumors were grouped according to the proportion of high-grade histology: <40%, 40–90%, and >90% (*p* < 0.001). Notably, the neuroendocrine component appeared to have no prognostic significance, regardless of whether it was present in low- or high-grade tumor areas [16].

In their case presentations, Falk, Ibrahim, and Gilmore use the term “goblet cell carcinoma (GCC) of the appendix”, emphasizing that it represents a distinct pathological entity and constitutes a diagnostic challenge for pathologists. The authors also highlight the need for further investigations to improve understanding of these rare and heterogeneous tumors [17,18,19].

In a Danish cohort study comprising 83 cases, Olsen et al. conducted a comprehensive analysis of GCC from demographic, pathological, prognostic, therapeutic, and survival perspectives. The study applied both the Tang histopathologic classification, confirming its prognostic relevance, and the TNM/AJCC staging systems, underscoring the complementary value of anatomical staging [20].

In 2018, Yozu et al. [22] proposed adopting the terminology “goblet cell adenocarcinoma (GCA)”, drawing a conceptual and biological parallel with colorectal adenocarcinoma. In their cohort of 126 patients, they validated the use of previously proposed histologic grading systems as effective tools for prognostic stratification [22].

Although the World Health Organization (WHO) reclassified goblet cell carcinoma (GCC) as goblet cell adenocarcinoma (GCA) in 2019, several studies published after this date continue to use the earlier terminology.

Zakka et al. conducted a retrospective study including 619 cases of GCC, identified through the National Cancer Database (NCDB) using the ICD-O-3 morphology code 8243/3 and topographic code C18.1 [23].

In a 2021 study, Webb et al. analyzed data from the NCDB, including patients diagnosed with appendiceal cancer between 1998 and 2012. They demonstrated that nodal involvement in appendiceal tumors varies in both incidence and prognostic impact, and may influence response to systemic therapy. Of the 14,918 cases of appendiceal tumors identified, 4435 were classified as goblet cell tumors, including adenocarcinoids and neuroendocrine carcinomas [24].

Another large retrospective cohort based on the SEER database was published by Wang G et al. in 2021 [25], reporting 1087 cases of GCC. This study aimed to evaluate the effect of chemotherapy in GCC patients, stratifying cases according to TNM staging [25].

In a Canadian retrospective study, Jedrzkiewicz et al. reported 24 cases of appendiceal neoplasms labeled as GCC or ex-GCC, emphasizing the importance of reclassification, which often leads to changes in clinical management. The authors further advocated for subspecialty pathology review of appendiceal tumors referred to specialized centers, given the implications for treatment planning [26].

The term goblet cell adenocarcinoma (GCA) has increasingly been adopted in the reporting of smaller clinical series, reflecting alignment with the WHO fifth edition classification.

In a 2023 retrospective study, Chen et al. evaluated appendectomy specimens and demonstrated that the classification of 40 GCA cases correlated well with the three-tiered histologic grading system (G1, G2, G3), supporting its utility in routine diagnostic practice [27].

In a molecular profiling study published in 2020, Arai et al. examined 495 appendiceal tumor specimens, identifying 53 cases of GCC [28]. Using next-generation sequencing (NGS) targeting a 592-gene panel alongside immunohistochemistry (IHC), they found that GCCs exhibited distinctly different mutational profiles compared to both appendiceal adenocarcinomas and neuroendocrine tumors. The authors emphasized that a deeper understanding of these molecular features may be critical for the development of targeted therapies and improved treatment strategies in GCC [28].

The most recent series was reported by Taniguchi et al. in 2025 [29], based on 39 GCA cases retrieved from the Japanese nationwide Center for Cancer Genomics and Advanced Therapeutics (C-CAT) database. This retrospective genomic study focused on advanced appendiceal carcinoma (AC) and its mutational landscape, revealing the following mutation frequencies: TP53 (5.2%), GNAS (5.4%), KRAS (2.5%), SMAD4 (50%), and wild-type tumors (31.4%). Their findings suggest that specific genetic alterations influence both clinical outcomes and chemotherapy responsiveness in GCA [29].

### 3.2. Tratment Patterns (Table 2)

#### 3.2.1. Surgical Treatment

Several authors advocate for prophylactic right hemicolectomy in order to reduce the risk of recurrence. Rossi conducted a study on a cohort of 48 patients (TNM stage I–II: 27 patients; stage III: 15 patients; stage IV: six patients). The median follow-up duration was 44 months and was complete for all patients. Acute appendicitis was the presenting symptom in 68.8% of cases. A total of 44 out of 48 patients initially underwent appendectomy, followed by prophylactic right hemicolectomy in 41 patients. Disease recurrence occurred in 10 of the 48 patients, with a median time to recurrence of 28 months (range: 4–159 months) [16].

**Table 2:** Included reports in treatment patterns.

No	Authors	Year	Title	Case Number	Grade	Stage	Surgery	Oncology	Type	Survival
1	Lamarca.et al. [15]	2019	Adenocarcinoma Ex-Goblet Cell: a Retrospective Experience.	23	A-0 B-14 C-9	IV-17	RHC-8 RHC+HIPEC-5	PC-17	FOLFOX/FOLFIRI	NR NR mOS 32.9mo
2	Nonaka et al. [16]	2015	Appendiceal Goblet cell tumors (GCTs) are clinically more aggressive, and have a worse outcome than midgut neuroendocrine tumors (mNETs).	48 (44a)		I/II-27 III-15 IV-6	A-44 pRHC-41 CRS-24	AC-9 PC-5	FOLFOX/CAPOX/FOLFIRI	1yOS-92% 3yOS-62.5% 5yOS-42%
3	Ibrahim et al. [18]	2020	Clinicopathological analysis of appendiceal goblet cell adenocarcinoma with peritoneal metastasis: World Health Organization grade predicts survival following cytoreductive surgery with intraperitoneal chemotherapy.	63 (48 GCA)	A-4 B-25 C-34	I–III-5 IV-58	RHC+HIPEC-55	AC-51	FOLFOX 4 FOLFIRI	G1/2mOS-98mo G3mOS-33mo
4	Wang et al. [25]	2021	Is adjuvant chemotherapy beneficial for stage II–III goblet cell carcinoid/goblet cell adenocarcinoma of the appendix?	619	H-360 L-259	II-512 III-107	RHC-603 TC-16	AC-99 No-496 NA-21		5yOS-85, 5/82.7/42.8% II-5yOS-100%/90.9% III-77.1
5	Das et al. [34]	2019	Treatment patterns and outcomes in goblet cell carcinoid tumors of the appendix.	2552	L-449 H-144 NA-830	I-279 II-811 III-152 IV-181	A-639 RHC-1288	AC-409		5yOS-77.8% 10yOS- 58.7%
6	Zakka et al. [23]	2016	Appendiceal Goblet Cell Carcinoids: Management Considerations from a Reference Peritoneal Tumour Service Centre and ENETS Centre of Excellence. Neuroendocrinology.	74	A-33 B-31 C-5 NA-5	I-2 II-29 III-12 IV-28	A-9 A+RHC-24 A+RHC+HIPEC-10 A+RHC+HIPEC+CRS-15 MS-15 PS-10	AC-18 PC-24	FOLFOX/FOLFIRI	I-5yOS-100% II-5yOS-87% III-5yOS-40% IV-5yOS-18%
7	Chen et al. [27]	2020	Outcomes in Peritoneal Carcinomatosis from Appendiceal Goblet Cell Carcinoma Treated with Cytoreductive Surgery and Hyperthermic Intraperitoneal Chemotherapy (CRS/HIPEC)	27	G1-17 G3-3		A-11 RHC-10 CRS/HIPEC-20	NAC-7 AC-17	FOLFOX BEVACIZUMAB (2)	1yOS-100% 3yOS-40% 5yOS-40%
8	Höfler et al. [38]	2018	Effects of Cytoreductive Surgery and Hyperthermic Intraperitoneal Chemotherapy (HIPEC) in the Treatment of Goblet Cell Carcinoma: A Prospective Cohort Study.	48	LD-6 PS-8 EID-7		RHC-6 pHIPEC+CRS-8 HIPEC+CRS-27 PS-7	AC-16 PC-19 SC-2	FOLFOX/CAPOX	LD-5yOS- 100% PS-5yOS-63% EID-5yOS- 57%
9	Madsen et al. [32]	2021	Appendiceal tumors with glandular and neuroendocrine features exhibiting peritoneal metastases—Critical evaluation of outcome following cytoreductive surgery with perioperative chemotherapy.	47	A-6 B-25 C-13		CRS-47 CRS+ICT-32	NAT-32 AC-21		mOS-48.5mo 5yOS-34.88% 10yOS-8.72%
10	Kowalsky et al. [30]	2015	Goblet cell carcinoids: characteristics of a Danish cohort of 83 patients.	83	A-34 B-40 C-9	I-12 II-35 III-3 IV-27	A-4 A+RHC-53 A+RHC+BSO-16 A+BSO-10 CRS+HIPEC-4	AC-24	SCLC r-11 CRC r-3 NET r-1 OC r-1	mOS-83mo 1yOS-90% 3yOS-58% 5yOS-38%
11	Palmer et al. [37]	2018	Outcomes of Surgical and Chemotherapeutic Treatments of Goblet Cell Carcinoid Tumors of the Appendix. Oncol.	86	A-48 B-21 C-10	I–III-67 IV-19	A-40 RHC-51 MOR+HIPEC-9	AC-9	5FU-5 FOLFOX-4	5yOS-68%
12	Adsay et al. [39]	2016	Curative Surgical Resection as a Component of Multimodality Therapy for Peritoneal Metastases from Goblet Cell Carcinoids.	43	A-4 B-22 C-7		CRS+HIPEC-43	NAT-32 AC-20	-	mOS-22mo 3yOS-39% 5yOS-9%
13	Clift et al. [8]	2018	Goblet cell carcinomas of the appendix: rare but aggressive neoplasms with challenging management.	21	A-8 B-10 C-3	I-1 II-10 III-5 IV-5	A-12 RHC-6+8 A+BSO-2 RHC+BSO-1 HIPEC-1	AC-6		1yOS-79.4% 3yOS-60% 5yOS-60%

Other authors also consider right hemicolectomy to remain the standard of care. In a study by Tsang, 86 patients diagnosed with goblet cell carcinoma (GCC) between 1984 and 2014 were identified through the British Columbia Cancer Agency and the Vancouver Lower Mainland Pathology Archive. Among the 67 patients with stage I–III disease, 51 underwent complete hemicolectomy, and nine received adjuvant chemotherapy based on 5-fluorouracil. In this population-based cohort, excellent survival outcomes were demonstrated for patients with stage I–III GCC and clinically apparent appendicitis. The median overall survival (OS) was not reached in patients with stage I–III disease [37].

Conversely, some studies have shown that complete right hemicolectomy does not significantly impact recurrence rates or disease-free survival. Lamarca conducted a retrospective single-center study involving 74 patients with GCC treated between 1996 and 2014. Of these, 76% received surgical intervention [including surgery alone (36%), cytoreductive surgery (CRS) combined with hyperthermic intraperitoneal chemotherapy (HIPEC) (36%), adjuvant chemotherapy (20%), and a combination of CRS and HIPEC followed by adjuvant chemotherapy (9%)]. A total of 23% of patients had advanced-stage disease managed with palliative treatment (chemotherapy or supportive care). Complete right hemicolectomy was performed in 64% of patients who underwent surgery. Disease-free survival (DFS) in patients receiving curative-intent treatment and progression-free survival (PFS) in those with palliative-intent treatment were 52.1 months (95% CI: 29.4–90.3), 75.9 months (95% CI: 26.6–not reached), and 5.3 months (95% CI: 0.6–5.7), respectively [23].

Some authors have highlighted that in cases of T3/T4 tumors, a significant benefit in 5-year survival was observed in patients undergoing colectomy compared to appendectomy (85.4% vs. 82.0%, *p* = 0.028). In the study by Kowalsky SJ, 1083 patients with goblet cell carcinoma (GCC) were included, of whom 81.8% were treated with right hemicolectomy. Patients who underwent hemicolectomy had more advanced T-stage tumors (66.6% T3 and 14.4% T4 vs. 55.8% T3 and 8.1% T4, *p* < 0.001). Surgical margins after appendectomy were positive in 17.3% of cases compared to 3.4% following hemicolectomy (*p* < 0.001). Among patients with T3/T4 tumors, there was a trend toward improved survival with right hemicolectomy (hazard ratio [HR] 0.42, *p* = 0.068). Omission of right hemicolectomy may be considered for select patients with T1/T2 GCC and negative appendectomy margins, given the low rates of nodal metastasis and lack of demonstrated survival benefit [40].

Other authors consider that cytoreductive surgery (CRS) combined with hyperthermic intraperitoneal chemotherapy (HIPEC) should be regarded as the primary treatment option for patients with peritoneal carcinomatosis (PC) arising from appendiceal goblet cell adenocarcinoma (GCA). Zambrano-Vera analyzed a prospective institutional database comprising 391 patients treated with CRS/HIPEC for appendiceal carcinomatosis between 1998 and 2018. Twenty patients with GCA were identified. All had undergone prior surgical intervention. Seven patients (35%) had received chemotherapy previously, with a median of 5 cycles (range: 3–8). The median peritoneal carcinomatosis index (PCI) was 6 (range: 1–39). Complete cytoreduction was achieved in 95% (19/20) of patients. The median follow-up period was 97 months. Overall survival (OS) rates at 1, 3, and 5 years were 100%, 74%, and 67%, respectively. Progression-free survival (PFS) rates at 1, 3, and 5 years were 94%, 67%, and 59%, respectively [27].

Other authors argue that long-term survival can be achieved in patients with peritoneal dissemination or those at high risk of developing peritoneal spread through CRS combined with HIPEC. Madsen reported the results of a study involving 48 patients with goblet cell carcinoma (GCC) treated at the European Neuroendocrine Tumor Center, Aarhus University Hospital, between 2009 and 2016. Patients with localized disease underwent right hemicolectomy. Those with peritoneal dissemination who met inclusion criteria for CRS plus HIPEC, as well as patients with high-risk features for developing peritoneal spread, received CRS plus HIPEC. Patients with disease deemed too extensive for surgery were offered palliative chemotherapy. Overall survival (OS) among six patients with localized disease and eight patients considered at risk for peritoneal dissemination was 100% after a median follow-up of 3.5 years. Among 27 patients with peritoneal dissemination eligible for CRS plus HIPEC, the median OS was 3.2 years, with a five-year survival rate of 57%. In contrast, median OS for seven patients with disease considered too extensive for surgery was 1.3 years (95% CI: 0.6–2.0), with a three-year survival rate of 20% [38].

Other authors consider that in metastatic disease, outcomes remain poor, although multiorgan resection (MOR) with or without hyperthermic intraperitoneal chemotherapy (HIPEC) may improve survival. In the study by Tsang, which included 86 patients with goblet cell carcinoma (GCC), 19 patients were stage IV; among them, 10 (62.5%) received 5-fluorouracil-based chemotherapy, and 11 (61%) underwent multiorgan resection (MOR) with or without HIPEC. Low mitotic rate and MOR ± HIPEC were associated with improved two-year overall survival (OS), but only MOR ± HIPEC remained statistically significant in multivariate analysis (hazard ratio [HR] 5.4, 95% confidence interval [CI] 1.4–20.9; *p* = 0.015). The presence of metastases at presentation was the strongest predictor of overall survival, with a median OS of 16.2 months (95% CI 9.1–29) for patients with stage IV disease [37].

Other authors have shown that patients with Tang C tumors exhibit limited survival and are not ideal candidates for a surgical approach. A study presented by Radomski prospectively analyzed 43 patients with goblet cell carcinoma (GCC) treated between 2005 and 2013 with 50 CRS-HIPEC procedures for peritoneal metastases; most patients received systemic neoadjuvant and/or adjuvant chemotherapy. The majority of patients had Tang B GCC. The estimated median survival after surgery was 59, 22, and 13 months for patients with Tang A, B, and C tumors, respectively. In a multivariate Cox regression analysis, poor survival was associated with patients having Tang B or C gastroenteropancreatic carcinoma, those undergoing incomplete macroscopic resection, and those presenting with symptoms at the time of CRS-HIPEC. Patients with Tang B and C tumors demonstrated survival rates similar to or worse than those with high-grade (AJCC grade 3) disseminated appendiceal mucinous neoplasms [39].

#### 3.2.2. Systemic Chemotherapy

Several studies suggest that chemotherapy may play an important role in this disease, both as adjuvant treatment (FOLFOX) and in the metastatic setting (FOLFOX/FOLFIRI). Das conducted a retrospective analysis of 23 patients with appendiceal goblet cell carcinoma (AGCC), of whom 17 were pathologically confirmed stage IV disease at Vanderbilt University Medical Center and treated with FOLFOX/FOLFIRI chemotherapy, either as adjuvant therapy (eight patients) or palliative therapy (17 patients). Right hemicolectomy (RHC) was performed in eight patients, and RHC combined with HIPEC in five patients. The median overall survival was 32.9 months [15].

Systemic chemotherapy regimens used for colorectal adenocarcinoma are indicated for advanced or recurrent disease and have shown encouraging results. Rossi studied a cohort of 48 patients (TNM stage I–II: 27; stage III: 15; stage IV: six). Forty-four of 48 patients initially underwent appendectomy, followed by prophylactic right hemicolectomy in 41 patients. Systemic adjuvant chemotherapy (FOLFOX/FOLFIRI) was administered to nine out of 48 patients with stage III disease following right hemicolectomy, and to five out of 48 patients with disseminated disease at diagnosis. The overall five-year survival rate was 41.6% [16].

Adjuvant chemotherapy (AC) was associated with improved overall survival (OS) in patients with stage III goblet cell adenocarcinoma (GCA), but not in those with stage II disease. The study by Zakka analyzed 619 patients with pathologic stage II and III GCA who underwent surgical resection between 2006 and 2015, identified from the National Cancer Database. AC was administered to 48 patients in stage II and 51 patients in stage III. Among stage II patients, the five-year OS did not differ significantly between those who received AC (96.9%) and those who did not (89.1%) (*p* = 0.236). In contrast, stage III patients who received AC had significantly improved five-year OS compared to those who did not (77.1% vs. 42.8%, *p* = 0.003) [25].

Another study did not identify any benefit from a specific adjuvant approach, although a treatment selection bias was observed. Lamarca conducted a retrospective single-center study including 74 patients with GCC between 1996 and 2014. Among these, 76% were treated surgically only (36%), with cytoreductive surgery (CRS) and hyperthermic intraperitoneal chemotherapy (HIPEC; 36%), adjuvant chemotherapy (20%), and a combination of CRS and HIPEC followed by adjuvant chemotherapy (9%). Twenty-three percent had advanced-stage disease suitable only for palliative treatment (chemotherapy or supportive care). FOLFOX chemotherapy was used in both adjuvant and palliative settings; safety profiles were as expected, and a high disease control rate (60%) was observed in the palliative cohort. The estimated median OS (all patients), disease-free survival (DFS; patients with curative intent), and progression-free survival (PFS; patients with palliative intent) were 52.1 months (95% CI: 29.4–90.3), 75.9 months (26.6–not reached), and 5.3 months (0.6–5.7), respectively [23].

Other authors have noted no clear benefit of systemic or regional perioperative chemotherapy. In the study by Barrak et al., 47 patients were included. The median OS for the entire cohort was 45 months, with 34.88% and 8.72% of patients surviving five and 10 years, respectively. The use of neoadjuvant chemotherapy or the type of chemotherapy administered did not demonstrate any impact on survival [35].

#### 3.2.3. Overall Survival

Several authors have demonstrated that the Tang classification is an independent prognostic factor for poor survival following multimodal therapy for goblet cell carcinoma (GCC).

A prospective study by Radomski analyzed 43 patients with GCC who underwent cytoreductive surgery (CRS) combined with hyperthermic intraperitoneal chemotherapy (HIPEC) between 2005 and 2013. The majority of patients had Tang B GCC. The estimated median survival time after surgery for patients with Tang A, B, and C GCC was 59, 22, and 13 months, respectively. In a multivariate Cox regression analysis, poor survival was associated with patients having Tang B or C goblet cell carcinoma. Patients with Tang A GCC demonstrated oncologic outcomes comparable to those with intermediate-grade disseminated appendiceal mucinous neoplasms (grade 2 American Joint Committee on Cancer [AJCC]), whereas patients with Tang B and C carcinoma exhibited survival rates similar or inferior to those with high-grade disseminated appendiceal mucinous neoplasms (grade 3 AJCC) [39].

In a retrospective study by Clift et al., 21 patients with GCC treated surgically at two tertiary referral centers were analyzed. There were 8, 10, and 3 tumors classified as Tang A, B, and C, respectively. The index surgery consisted of appendectomy (12), right hemicolectomy (6), or resections including appendix/right colon, omentum, and gynecological organs (3). Eight patients underwent a complete right hemicolectomy. Surgical interventions for recurrence included small bowel resection (2), tumor debulking with peritonectomy and heated intraperitoneal chemotherapy, as well as hysterectomy and bilateral salpingo-oophorectomy (1). The mean OS (and OS at one, three, and five years) for Tang A, B, and C tumors was 73.1 months (85.7%, 85.7%, 51.4%), 83.7 months (all 66.7%), and 28.5 months (66.7%, 66.7%, not reached), respectively [8].

Olsen’s study included 83 patients with goblet cell carcinoma (GCC), of whom 54 had localized appendiceal disease (female/male: 29/25). According to the TNM classification, 24% were stage I, 70% stage II, and 6% stage III. All patients underwent surgical intervention. Sixty-three patients (76%) had radical resections, including all patients with localized disease. According to the Tang classification, median OS for groups A, B, and C was 118, 83, and 20 months, respectively (*p* = 0.0002) [30].

Conversely, another study did not find prognostic significance for the Tang classification. Shyu et al. analyzed 63 patients with peritoneal metastases of GCC treated with cytoreductive surgery and hyperthermic intraperitoneal chemotherapy (CRS-HIPEC), stratified by the 5th edition WHO grading and the Tang classification schemes. The majority (73%) of peritoneal metastases were WHO grade 3 (G3), with fewer cases of grade 2 (G2) (16%) and grade 1 (G1) (11%). No significant differences in overall survival were observed between tumors of the three Tang grades, whereas, the WHO 5th edition GCC grade was a clinically relevant independent predictor of survival in patients treated with CRS-HIPEC [36].

Other authors demonstrated that the presence of metastases at diagnosis was the strongest predictor of overall survival. In Tsang’s study, which included 86 patients diagnosed with GCC, 19 were stage IV. Ten patients received 5-fluorouracil-based chemotherapy, and 11 underwent multiorgan resection (MOR) ± HIPEC. Low mitotic rate and MOR ± HIPEC were associated with improved two-year OS, however, only MOR ± HIPEC remained significant in multivariate analysis (hazard ratio 5.4, 95% confidence interval 1.4–20.9; *p* = 0.015). Median OS for stage IV patients was 16.2 months (95% CI 9.1–29) [37].

### 3.3. Critical Appraisal

1. This is an extremely rare pathological entity, and much of the literature consists of case reports or small case series originating from North America, Europe, or Asia. However, these sources are not uniform in terms of nomenclature. AGCA remains a rare neoplasm globally, with the best-documented incidence data coming from North American databases.The lack of standardized reporting and variable diagnostic criteria across regions contribute to an epidemiological gap that warrants multinational registry collaboration (Table S2) and uniform adoption of WHO and Tang/Yozu classifications.
2. Most retrospective studies have been conducted on large cohorts, predominantly from North American populations, based on SEER and NCDB databases. These studies often include cases reported prior to 2019—before goblet cell tumors (GCTs) were reclassified as goblet cell adenocarcinomas (GCAs). Consequently, nomenclature changes were not reflected in earlier publications.
3. Therefore, even in studies published after 2019, the term “GCC” is still frequently used. Although “GCC” (goblet cell carcinoid) had long been the preferred term in the literature, its inclusion of “carcinoid” has caused confusion with well-differentiated neuroendocrine tumors (NETs), potentially leading to misdiagnosis and inappropriate treatments. Despite Yozu’s 2018 [22] recommendation for reclassification to GCA, and the WHO’s formal adoption of this nomenclature in 2019, inconsistency persists. Thus, we observe a significant source of confusion in referring to the same pathological entity by different names. At present, approximately 20 studies have adopted the 2019 WHO classification using the term GCA in case reports, small series, and larger cohorts.
4. The diagnosis of GCA was confirmed based on its characteristic histopathological features, and the non-endocrine component was verified through immunohistochemistry (IHC), although this may erroneously lead pathologists toward a NET diagnosis. Therefore, subspecialty pathology review of appendiceal neoplasms at referral centers is warranted, as emphasized by Jedrkiewicz [25].
5. In most articles, the Tang classification—a histological grading system that more accurately reflects tumor biology and behavior—is used alongside the TNM classification—a universal anatomical staging framework critical for treatment planning and prognostication. The integration of both systems in diagnosing GCA offers a clearer understanding of tumor aggressiveness, the need for adjuvant treatment, and the surgical extent required. Additionally, consistent use and reporting of TNM staging is recommended to standardize cancer registry data across institutions.
6. In an effort to further characterize this entity, molecular studies have also been conducted. Two major studies concluded that GCA presents a mutational profile distinctly different from both appendiceal adenocarcinomas and neuroendocrine tumors. Molecular classification was described in two large retrospective series based on analysis of resected appendiceal carcinoma specimens, aimed at identifying prognostic and predictive mutations to support potential targeted therapies [35,40].
7. The lack of randomized controlled trials has led to conflicting data regarding the survival benefits of various surgical approaches. While some authors advocate for standard right hemicolectomy (RHC), others, such as Lamarca [23], report no survival advantage. In contrast, Tsang considers RHC to be the standard therapy, while Rossi supports it as a preventive strategy against recurrence. Several authors emphasize the importance of addressing peritoneal disease through cytoreductive surgery (CRS) and hyperthermic intraperitoneal chemotherapy (HIPEC), noting the marked peritoneal tropism of GCA. CRS and HIPEC are strongly supported for patients with peritoneal involvement by authors such as Madsen and Tsang, whereas others, including Radomski, suggest that patients with Tang C tumors do not benefit significantly from aggressive surgical approaches.
8. There is also no consensus regarding systemic chemotherapy. Some authors advocate for diagnostic laparoscopy followed by neoadjuvant chemotherapy [32]. Most adopt gastrointestinal regimens (e.g., FOLFOX or FOLFIRI) [15,16], although others have applied chemotherapy protocols typically used for ovarian, pulmonary, or neuroendocrine tumors [30]. The benefit of adjuvant chemotherapy in stage III disease is supported by Zakka, yet other studies [23,32] fail to confirm this, possibly due to small sample sizes or cohort heterogeneity. In selected cases, targeted therapies have been proposed when actionable molecular markers or receptors are identified [27].
9. Overall survival (OS) rates remain highly variable, even when applying the same Tang classification system. This variation is attributed to differences in cohort composition and limited patient numbers. For example, Clift et al. [8] reported median OS for Tang A, B, and C tumors of 73.1 months, with 1-year OS rates of 85.7%, 85.7%, and 51.4%, respectively—lower than figures reported by Olsen. While some studies support the prognostic value of Tang grading, with OS rates of 118, 83, and 20 months for classes A, B, and C, respectively (*p* = 0.0002) [30], others, such as Shyu, found no statistically significant difference in OS among the Tang subtypes [36].
10. This scarcity of high-level evidence complicates the establishment of standardized treatment protocols and hinders the ability to perform evidence-based comparisons of surgical techniques, chemotherapy regimens, or emerging targeted therapies. Consequently, clinical decisions are often extrapolated from guidelines for colorectal adenocarcinoma or neuroendocrine tumors, which may not fully capture the distinct biological behavior of GCA. Addressing this knowledge gap through multicenter registries and collaborative prospective trials is essential to improve outcomes and develop consensus-based treatment strategies.
11. Nevertheless, several treatment recommendations can be made:

Right hemicolectomy is generally recommended both for localized disease and in the presence of regional intraperitoneal spread, in order to reduce the risk of metastatic progression.In cases of disseminated disease at presentation, including peritoneal carcinomatosis, primary cytoreductive surgery followed by intraperitoneal or systemic chemotherapy may be considered, ideally within a clinical trial setting.

Adjuvant chemotherapy should be considered in patients with positive lymph nodes or other high-risk features such as large tumor size or perforation.

FOLFOX as first-line and FOLFIRI as second-line regimens remain the most commonly utilized therapeutic protocols.

### 3.4. Guideline Discrepancies in the Management of Appendiceal Goblet Cell Adenocarcinoma (AGCA)

The management of appendiceal goblet cell adenocarcinoma (AGCA) reveals marked discrepancies between major international guideline bodies, particularly the National Comprehensive Cancer Network (NCCN) and the European Neuroendocrine Tumor Society (ENETS).

1. The NCCN Guidelines tend to align AGCA with appendiceal or colorectal adenocarcinomas, advocating for aggressive surgical management strategies, such as right hemicolectomy for localized disease, and systemic chemotherapy regimens similar to those used in colorectal cancer. This approach reflects the high-grade behavior and aggressive clinical course typically observed in AGCA.
2. In contrast, the ENETS Guidelines, which primarily address neuroendocrine neoplasms, frequently classify AGCA under the broader category of mixed neuroendocrine-nonneuroendocrine neoplasms (MiNENs). ENETS emphasizes a multidisciplinary approach that includes aspects of neuroendocrine tumor management, such as consideration of somatostatin analogs, proliferation indices, and personalized systemic therapy regimens tailored to the tumor’s neuroendocrine component.

This divergence in classification and therapeutic strategy leads to significant variability in clinical practice, particularly regarding the extent of surgery and choice of systemic treatment. The absence of universally accepted, disease-specific guidelines highlights the critical need for dedicated prospective research and the development of harmonized international protocols for AGCA.

## 4. Discussions

Primary malignant tumors of the appendix are rare, with goblet cell adenocarcinoma (GCA) accounting for less than 5% of cases. GCA is a distinct neoplasm characterized by amphicrine or biphenotypic differentiation, combining glandular/mucinous and neuroendocrine features—an aspect that has contributed to multiple historical and proposed nomenclatures [41,42,43,44].

Several terminologies, including goblet cell carcinoid, crypt cell carcinoma, microglandular carcinoma, adenocarcinoid, mixed adenoneuroendocrine carcinoma (MANEC), amphicrine carcinoma, mixed adenocarcinoid carcinoid, and mucinous carcinoid, have caused diagnostic confusion around this entity. In one study, goblet cell tumors were the second most common type after low-grade appendiceal mucinous neoplasms, potentially reflecting their uncommon incidence and evolving classification systems [45].

Goblet cell carcinoid tumors represent a heterogeneous group of appendiceal neoplasms with unique histopathological features. Although their pathogenesis is not entirely understood, they are believed to originate from the basal pluripotent stem cells of the intestinal epithelial crypt. The loss of Notch signaling is considered the primary driver mutation, promoting tumor progression and conferring a clinical course similar to poorly differentiated adenocarcinomas with minimal neuroendocrine differentiation [46].

First described in 1969 by Gagné et al. as an appendiceal tumor exhibiting both adenocarcinoma and carcinoid features [44], the term “goblet cell carcinoid” (GCC) was later introduced in 1974 by Subbuswamy et al. [45]. Warkel et al., in 1978, described GCC as an aggressive adenocarcinoid subtype with an intermediate prognosis between carcinoid tumors and adenocarcinomas [46].

The 2010 WHO classification of digestive system tumors introduced the term “mixed adenoneuroendocrine carcinoma” (MANEC) for these tumors. However, this term has proven misleading, as it suggests the presence of both a neuroendocrine carcinoma and a conventional adenocarcinoma component, whereas goblet cell tumors exhibit true amphicrine differentiation [47].

Wen et al. emphasized the diagnostic challenges posed by goblet cell tumors, showing that the term “carcinoid” led many pathologists to incorrectly stage these tumors using neuroendocrine tumor (NET) protocols, including the Ki67 proliferation index. Moreover, they noted that the label MANEC was often misinterpreted by oncologists as indicating a neuroendocrine carcinoma, resulting in the inappropriate recommendation of platinum-based chemotherapy [48].

Such misinterpretations have contributed to variability in diagnosis and reporting. Accurate classification is clinically significant for both prognostic prediction and treatment planning. Another study pointed out that multiple classification systems have caused difficulty for pathologists unfamiliar with this rare entity [37]. Due to inconsistencies in terminology, survival estimates, and treatment recommendations, several studies have attempted to clarify the pathology of GCA [16].

Ultimately, the tumor’s genetic profile has been delineated, demonstrating distinct morpho-molecular characteristics that differentiate it histologically and genetically from colorectal-type appendiceal adenocarcinomas. Alterations in genes associated with Wnt signaling—excluding APC—may act as tumorigenic drivers. The absence of KRAS/NRAS mutations in some cases suggests eligibility for targeted anti-EGFR therapies [49].

In 2019, the World Health Organization (WHO), in the fifth edition of the Digestive System Tumors Classification, introduced the updated term appendiceal goblet cell adenocarcinoma (AGCA), reflecting the tumor’s adenocarcinomatous nature and advising it be managed as an adenocarcinoma [1]. Thus, what was once termed goblet cell carcinoid is now more accurately classified as appendiceal goblet cell adenocarcinoma.

The latest (ninth edition, 2022) AJCC staging manual for primary appendiceal carcinomas acknowledges this shift, officially adopting the term goblet cell adenocarcinoma to replace “goblet cell carcinoid” [50]. This clarification is essential, as GCAs require more aggressive management and surveillance than appendiceal neuroendocrine tumors (NETs) [51].

- **Diagnostic pitfalls**: Goblet cell adenocarcinoma (GCA) often presents with overlapping histological features of both adenocarcinoma and neuroendocrine tumors, which can lead to misclassification as typical carcinoid or mixed adenoneuroendocrinecarcinoma (MANEC). The diagnosis requires awareness of the dual phenotype, with both glandular (mucin-secreting) and neuroendocrine features, best demonstrated by morphology and immunohistochemistry (e.g., synaptophysin/chromogranin A positivity with Ki-67 > 20% in high-grade cases).
- **Differentiation from MANEC and typical carcinoids**: According to the 2019 WHO classification, GCAs are considered distinct from MANECs, as they represent a unique histologic and molecular entity. Unlike classical NET G1/G2 carcinoids, GCAs tend to be more aggressive, display infiltrative growth, and have a higher proliferative index.
- **Imaging and tumor markers for surveillance**: We have also added comments on surveillance strategies. While CT and MRI remain the primary modalities for post-treatment follow-up, especially in high-grade or disseminated disease, tumor markers such as CEA, CA19-9, and occasionally chromogranin A may be useful for monitoring, although no marker is specific. Their utility is often case-dependent and guided by initial expression levels.

A literature review via PubMed has shown a steady increase in reported GCA cases over the past decade, likely reflecting improved diagnostic criteria and greater awareness among pathologists [52]. The age of our patient is consistent with the literature, which cites a median age of 53 years (range 47–59), with no gender predilection [14,52].

Preoperative diagnosis is rare, occurring in fewer than 1% of cases [42]. Most diagnoses occur incidentally during appendectomy or in the context of acute appendicitis, or at advanced tumor stages, observed in 10–63% of cases [14,43]. Known for their peritoneal dissemination potential [53], these tumors often require extensive preoperative imaging and endoscopic assessment.

Because symptoms such as right lower quadrant (RLQ) pain, palpable mass, and inflammatory markers are nonspecific, diagnosis typically begins with imaging. CT often reveals an enlarged appendix with thickened walls, typically interpreted as acute appendicitis. Thus, appendiceal malignancies are frequently underestimated, with appendectomy as the initial intervention. Histopathological diagnosis then dictates further therapy. However, this approach may subject patients to multiple surgeries and psychological distress [14].

In our case, imaging and abdominal tumor syndrome findings prompted a full endoscopic workup. Colonoscopy was unremarkable except for a 0.5 cm cecal invagination at the appendix base with normal-appearing mucosa. Biopsies revealed acellular mucin without dysplasia or malignancy, raising suspicion for a mucinous neoplasm and suggesting an epithelial malignancy of appendiceal origin. A right hemicolectomy was thus indicated.

Right hemicolectomy is the preferred approach for localized disease, endorsed by multiple international guidelines, although a clear survival benefit is not uniformly demonstrated in stage I–III tumors [38]. Nevertheless, guidelines from ASCRS, NANETS, and ENETS recommend colectomy with regional lymphadenectomy in all GCA cases, citing a high rate of nodal metastases [7,54,55]. The Japanese Classification of Colorectal Carcinoma likewise advocates for right ileocolectomy in appendiceal carcinoma [56].

In emergent cases diagnosed post-appendectomy, further surgical intervention remains debated. NCCN guidelines recommend right hemicolectomy with at least 12 lymph nodes examined for proper staging, particularly in tumors with lymphovascular invasion or positive margins [57]. Our case involved adequate lymphadenectomy, with two macrometastatic nodes among 29 resected.

For metastatic disease, cytoreductive surgery (CRS) with hyperthermic intraperitoneal chemotherapy (HIPEC) is an option, though treatment protocols vary across centers. Despite the lack of randomized trials due to rarity, institutional series suggest improved disease-free and overall survival with CRS-HIPEC when performed in specialized centers [29,58]. Diagnostic laparoscopy is often used to assess feasibility.

CRS-HIPEC suitability is based on peritoneal cancer index (PCI), histologic subtype, KRAS status, and performance status. In high-grade Tang B/C tumors or those with carcinomatosis, HIPEC may prolong survival and improve local control [1,59].

Systemic chemotherapy offers added survival benefit, especially in node-positive disease [34,58,59,60]. A recent multicenter study (ESMO Sarcoma and Rare Cancers Congress 2023) showed that fluoropyrimidine and oxaliplatin-based chemotherapy improved outcomes in node-positive patients, with median survival not reached [61]. NCCN guidelines recommend treating GCA according to protocols for colonic adenocarcinoma [57].

In our case, carcinomatosis discovered intraoperatively prompted extended surgery (omentectomy, bilateral adnexectomy, resection of peritoneal nodules) and adjuvant chemotherapy per colorectal protocols. Prognosis is closely tied to tumor stage and grade. Most low-grade cases are stage I–II, though one-third develop metastases. In contrast, 50–70% of high-grade cases are stage IV [1].

Metastases frequently affect the peritoneum, omentum, abdominal wall, and ovaries. Survival ranges from 84–204 months in low-grade tumors and 60–86 months in intermediate-grade cases. High-grade, disseminated tumors carry worse prognoses (29–45 months), with 5-year survival reported at 40–60% [1,14]. Late recurrences, even 9 years post-resection, highlight the aggressive nature of GCA [13]. Adjuvant therapy is thus warranted in high-risk cases [55].

Given the presence of carcinomatosis, in our case initial surgery was palliative. After adjuvant chemotherapy, reoperation addressed residual carcinomatosis identified via PET-CT as a solitary “hot” nodule near the right iliac vein. Due to progression and confirmed peritoneal spread, second-line chemotherapy (irinotecan, capecitabine, bevacizumab) was initiated.

KRAS exon 2 mutation (G12D) ruled out anti-EGFR therapy, favoring bevacizumab (a VEGF-A inhibitor). This aligns with current guidelines recommending anti-angiogenic therapy in right-sided tumors or KRAS-mutated cancers [57,61]. Clinical trials support bevacizumab’s efficacy across tumor sites, reinforcing its use in this case.

Although limited by the rarity of AGCA, emerging data suggest several potential therapeutic targets. **KRAS mutations** have been identified in a subset of cases, supporting exploration of MAPK pathway inhibitors. Dysregulation of the **Wnt/β-catenin** and **Notch** signaling pathways may also play a role in tumor progression, though their therapeutic implications remain investigational. While most AGCA tumors are microsatellite stable and show limited response to **immune checkpoint inhibitors**, isolated reports suggest that **immunotherapy** may be beneficial in select high-grade or treatment-refractory cases. Further research, including molecular profiling and enrollment in rare tumor trials, is essential to better define the role of targeted and immune-based therapies.

There are notable deficiencies in standardized treatment for this entity, including variable guideline approaches, lack of specialized centers with expertise in this rare pathology, and an absence of dedicated clinical trials. Furthermore, the rarity of the disease limits institutional experience, and the diagnostic challenges from the outset further complicate management.

### Study Limitations

This study is subject to several limitations, primarily stemming from the rarity of appendiceal goblet cell adenocarcinoma (AGCA), which restricts the availability of large, prospective cohorts and limits statistical power. Historical inconsistencies in terminology—such as the interchangeable use of goblet cell carcinoid (GCC), mixed adenoneuroendocrine carcinoma (MANEC), and AGCA—have contributed to diagnostic confusion and potential misclassification in the previous literature. Treatment strategies remain non-standardized across institutions and guidelines (ASCRS, NANETS, ENETS, NCCN), with therapeutic decisions often extrapolated from colorectal adenocarcinoma protocols. Furthermore, the absence of randomized controlled trials, especially concerning systemic chemotherapy and HIPEC, hampers definitive conclusions on treatment efficacy. Preoperative diagnosis is exceedingly rare, complicating early therapeutic planning, while the limited availability of molecular profiling in routine clinical practice restricts the use of targeted therapies. Long-term outcome data and recurrence patterns are poorly defined, despite reports of late recurrence beyond five years. Finally, much of the current evidence derives from single-center experiences or retrospective series, which may not be generalizable to broader patient populations. These limitations underline the need for multicenter collaboration, prospective registries, and molecular characterization studies to optimize the diagnosis, staging, and management of AGCA.

## 5. Conclusions

Appendiceal Goblet Cell Adenocarcinoma (AGCA) is an exceptionally rare malignancy that presents significant diagnostic and therapeutic challenges for pathologists, surgeons, and oncologists alike. Characterized by its unique biological heterogeneity, AGCA displays both glandular (adenocarcinomatous) and neuroendocrine differentiation. Clinically, its presentation often mimics acute appendicitis, frequently resulting in initial management with suboptimal surgery—appendectomy. However, the disease carries a substantial risk of peritoneal dissemination and recurrence, particularly when diagnosed at advanced stages. The role of chemotherapy remains incompletely defined, and standardized therapeutic protocols are lacking due to the rarity of the condition and limited high-quality evidence. Recent updates introduced in the ninth edition of the AJCC staging system and endorsed by the World Health Organisation (WHO), as well as being reflected in current international clinical guidelines, now provide a more cohesive framework for the classification and management of AGCA. Given the histological and molecular heterogeneity of appendiceal neoplasms, accurate classification and comprehensive multidisciplinary evaluation are essential. AGCA should be managed analogously to high-grade appendiceal adenocarcinomas, integrating surgical, chemotherapeutic, and potentially targeted therapeutic modalities to optimize outcomes. Adoption of these guidelines facilitates multidisciplinary communication and enhances understanding of the tumor’s natural history, biological behavior, and prognosis.

## Data Availability

The datasets generated and/or analyzed during the current study are not publicly available due to privacy and ethical concerns, but are available from the corresponding author on reasonable request.

## Supplementary Materials

The following supporting information can be downloaded at: https://www.mdpi.com/article/10.3390/life15071047/s1, Figure S1: PRISMA flow chart for included reports in diagnosis challenges; Figure S2: PRISMA flow chart for included reports in treatment patterns; Table S1: Advanced search builder for appendiceal goblet cell adenocarcinoma and diagnosis/treatment; Table S2: Regional reporting incidence of appendiceal tumors.

## Abbreviations

GCA	Goblet Cell Adenocarcinoma
AGCA	Appendiceal Goblet Cell Adenocarcinoma
GCC	goblet cell carcinoid
GCT	goblet cell tumor
MiNEN	mixed neuroendocrine-nonneuroendocrine neoplasm
MANEC	mixed adenoneuroendocrine carcinoma
FOLFOX	fluorouracil, leucovorin calcium (folinic acid)and oxaliplatin
FOLFIRI	fluorouracil, leucovorin calcium (folinic acid) and irinotecan
WHO	World Health Organization
AJCC	American Joint Committee on Cancer
CRS	Cytoreductive Surgery
HIPEC	hyperthermic intraperitoneal chemotherapy
NET	Neuroendocrine Tumor
mNET	midgut Neuroendocrine tumors
CEA	Carcinoembryonic antigen
COPD	chronic obstructive pulmonary disease
FEV	Forced Expiratory Volume
IT	inspiratory time
CT	computed Tomography
HP	histopathology
IHC	Immunohistochemistry
OS	overall survival
CSS	cancer specific survival
PFS	progression free survival
NCDB	National Cancer Database
ICD-O	International Classification of Diseases for Oncology
SEER	Surveillance, Epidemiology, and End Results
AC	appendiceal carcinoma
a-	appendix
A-	appendectomy
NR	not reach
RHC	right hemicolectomy
MS	metastasectomy
PS	palleative surgery
AC	adjuvant chemotherapy
PC	palleative chemotherapy
SC	Supportive care
ICT	intraperitoneal chemotherapy
BSO	bilateral salpingo-oophorectomy
SCLC r	Small-cell-lung-cancer regimen
CRC r	Colorectal cancer regimen
NET r	Neuroendocrine Tumor regimen
OC r	ovarian cancer regimen
MOR	multiorgan resection
LD	localized disease,
PS	peritoneal spread
EID	extensive intraperitoneal disease
PCI	peritoneal carcinomatosis index
ENETS	European Neuroendocrine Tumor Society
ASCRS	American Society of Colon and Rectal Surgeons
NANETS	North American Neuroendocrine Tumor Society
NCCN	National Comprehensive Cancer Network
ESMO	European Society for Medical Oncology
