# Supplementary material for: High-Grade Appendiceal Goblet Cell Adenocarcinoma—A Literature Review Starting from a Rare Case
Source: Life (Basel). 2025 Jun 30;15(7):1047. doi: 10.3390/life15071047 (PMC12299544; doi:10.3390/life15071047)
Supplement: Supplementary file 1 [file life-15-01047-s001.zip › life-3673810-supplementary.pdf]

Table S1 Advanced search builder for appendiceal goblet cell adenocarcinoma and diagnosis/treatment

Search	Query	Results	Time
#10	Search: **appendiceal goblet cell adenocarcinoma treatment** Filters: **English, Adult: 19+ years, from 2015 - 2025**	92	04:13:20
#9	Search: **appendiceal goblet cell adenocarcinoma treatment** Filters: **English, from 2015 - 2025**	188	04:13:11
#8	Search: **appendiceal goblet cell adenocarcinoma treatment** Filters: **English**	473	03:36:42
#7	Search: **appendiceal goblet cell adenocarcinoma diagnosis** Filters: **English, Adult: 19+ years, from 2015 - 2025**	108	04:54:15
#6	Search: **appendiceal goblet cell adenocarcinoma diagnosis** Filters: **English, from 2015 - 2025**	218	04:54:06
#5	Search: **appendiceal goblet cell adenocarcinoma diagnosis** Filters: **from 2015 - 2025**	240	03:34:56
#4	Search: **appendiceal goblet cell adenocarcinoma diagnosis**	744	03:33:27
#3	Search: **appendiceal goblet cell adenocarcinoma**	1,122	03:27:35
#2	Search: **appendiceal goblet cell carcinoids**	1,069	03:26:37
#1	Search: **appendiceal tumors**	5,830	03:25:26

Table S2 Regional reporting incidence of appendiceal tumors

Database/Region	Incidence (per 100,000/year)	% of Appendiceal Tumors	Notes
SEER (U.S.)	0.05–0.3	6–14%	Increasing trend, better classification
NCDB (U.S.)	Similar to SEER	~7%	Covers 70% of U.S. cancer cases
Europe (varied sources)	<1 (estimated)	~5–10% (est.)	Sparse, not consistently disaggregated
Asia	Unknown/rare	Rare	Limited data, mostly case reports

Figure S1 PRISMA flow chart for included reports in diagnosis challenges

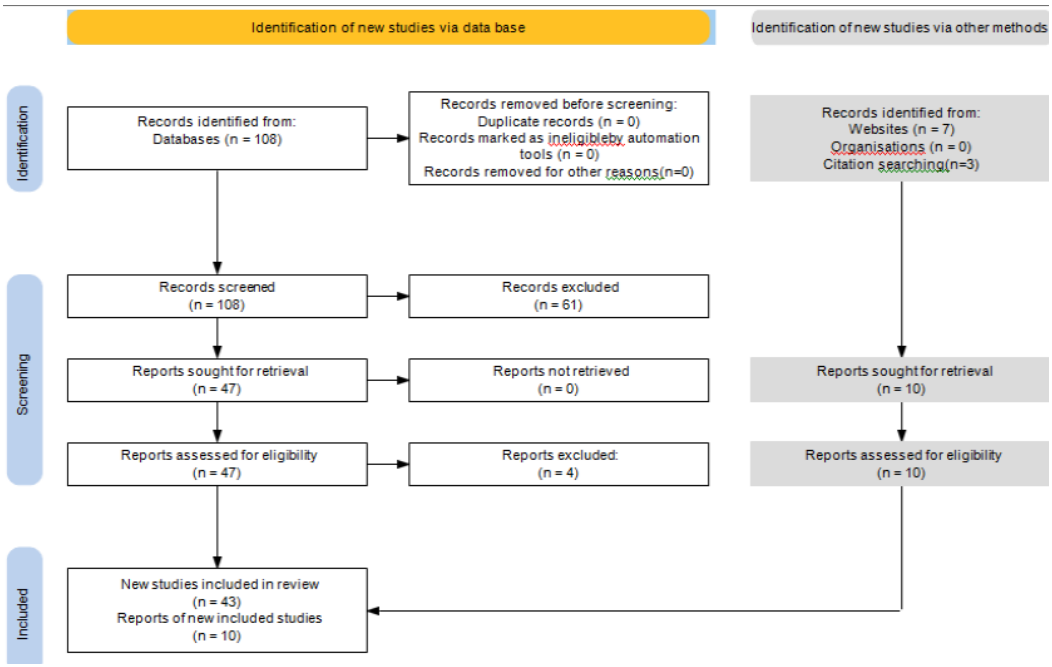

Figure S2 PRISMA flow chart for included reports in treatment patterns

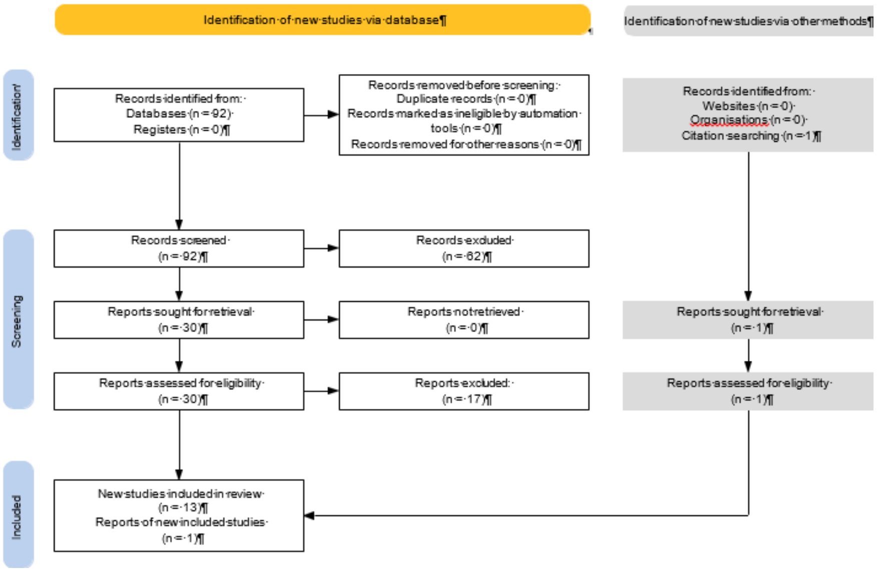

Identification of new studies via database
Identification of new studies via other methods
Identification
Records identified from: Databases (n = 92) Registers (n = 0)
Records removed before screening: Duplicate records (n = 0) Records marked as ineligible by automation tools (n = 0) Records removed for other reasons (n = 0)
Records identified from: Websites (n = 0) Organisations (n = 0) Citation searching (n = 1)
Screening
Records screened (n = 92)
Records excluded (n = 62)
Reports sought for retrieval (n = 30)
Reports not retrieved (n = 0)
Reports sought for retrieval (n = 1)
Reports assessed for eligibility (n = 30)
Reports excluded: (n = 17)
Reports assessed for eligibility (n = 1)
Included
New studies included in review (n = 13) Reports of new included studies (n = 1)
